# Genome-wide mutation profiles of colorectal tumors and associated liver metastases at the exome and transcriptome levels

**DOI:** 10.18632/oncotarget.4246

**Published:** 2015-06-01

**Authors:** Byungho Lim, Jihyeob Mun, Jeong-Hwan Kim, Chan Wook Kim, Seon Ae Roh, Dong-Hyung Cho, Yong Sung Kim, Seon-Young Kim, Jin Cheon Kim

**Affiliations:** ^1^ Medical Genomics Research Center, Korea Research Institute of Bioscience and Biotechnology (KRIBB), Daejeon, Republic of Korea; ^2^ Department of Functional Genomics, University of Science and Technology, Daejeon, Republic of Korea; ^3^ Department of Surgery, University of Ulsan College of Medicine, Seoul, Republic of Korea; ^4^ Institute of Innovative Cancer Research and Asan Institute for Life Sciences, Asan Medical Center, Seoul, Republic of Korea; ^5^ Graduate School of East-West Medical Science, Kyung Hee University, Gyeonggi-do, Republic of Korea

**Keywords:** colorectal cancer, exome sequencing, liver metastasis, RNA sequencing, somatic mutation

## Abstract

To characterize the mutation profiles of colorectal cancer (CRC) primary tumors (PTs) and liver metastases (CLMs), we performed both whole-exome and RNA sequencing. Ten significantly mutated genes, including *BMI1, CARD11*, and *NRG1*, were found in 34 CRCs with CLMs. We defined three mutation classes (Class 1 to 3) based on the absence or presence of mutations during liver metastasis. Most mutations were classified into Class 1 (shared between PTs and CLMs), suggesting the common clonal origin of PTs and CLMs. Class 1 was more strongly associated with the clinical characteristics of advanced cancer and was more frequently superimposed with chromosomal deletions in CLMs than Class 2 (PT-specific). The integration of exome and RNA sequencing revealed that variant-allele frequencies (VAFs) of mutations in the transcriptome tended to have stronger functional implications than those in the exome. For instance, VAFs of the *TP53* and *APC* mutations in the transcriptome significantly correlated with the expression level of their target genes. Additionally, mutations with high functional impact were enriched with high VAFs in the CLM transcriptomes. We identified 11 mutation-associated splicing events in the CRC transcriptomes. Thus, the integration of the exome and the transcriptome may elucidate the underlying molecular events responsible for CLMs.

## INTRODUCTION

CRC develops through a well-established sequence of events that are characterized by specific mutations: inactivating *APC* mutations lead to the development of a small benign adenoma; activating *KRAS* mutations are associated with the formation of a large adenoma; and diverse mutations in *TP53, PIK3CA*, and TGF-*β* pathway genes drive the evolution of a malignant carcinoma [1, 2]. Recent genome-scale studies identified additional frequent mutations in *ARID1A, CDH10, DOCK, FAM123B, FAT4*, and *SOX9* that might be responsible for CRC development [3, 4]. In addition, mutations overrepresented in CRC subgroups, which are characterized by microsatellite instability (MSI) or a CpG island methylator phenotype, were also reported [5, 6]. However, genetic alterations associated with metastasis are largely unknown, even though metastasis is the major cause of deaths from CRC.

Thus far, no recurrent metastasis-specific mutations have been demonstrated [7]. Instead, evidences have shown that PT-derived mutations may drive metastatic progression. In prostate cancer, the clonal populations that lead to distant metastases are represented within PTs [8]. In CRC, the genomic features between colorectal PTs and matched metastatic tumors were highly concordant [9], suggesting that the genetic alterations of metastatic tumors may descend from those of PTs. Thus, it is essential to monitor the identity of PT-derived mutations during metastasis.

Nevertheless, undoubted differences between PTs and CLMs might potentiate the existence of *de novo* metastasis-specific mutations. To understand these genetic alterations, recent studies have compared the mutational profiles between PTs and matched CLMs using targeted sequencing, cancer mini-exome sequencing, whole-exome sequencing, and whole-genome sequencing. However, a few groups performed targeted sequencing for only a subset of known cancer-associated genes [9–11], whereas the others sequenced only a small number of samples despite their genome-wide approaches [12, 13]. Hence, unbiased whole-exome analyses using sufficient sample sizes are required to determine whether CLM-specific mutations exist. In addition, a recent study showed that the integration of whole-exome sequencing with RNA sequencing facilitated more sensitive identification of cancer drivers and therapeutically targetable genes by providing greater mutation signal than the DNA in expressed mutations [14]. Therefore, we perform both whole-exome and RNA sequencing using tumor samples from Korean CRC patients with CLMs. We report the identity, pattern, and frequency of somatic mutations in the exomes and transcriptomes of PTs and CLMs.

## RESULTS

### Global molecular patterns of PTs and associated CLMs

To evaluate the somatic mutations found in PTs and CLMs, we performed both whole-exome sequencing (exome-seq) and RNA sequencing (RNA-seq) across a total of 57 tissues that encompass normal tissues, PTs, and CLMs from 19 CRC patients with CLMs (Supplementary Table S1). We used high-purity tumors (approximately > 90% tumor cell contents under histological examination) to improve mutation detection (Supplementary Figure S1A). However, tumor purity estimated mathematically using ASCAT v2.1 was lower than the pathology-based estimates (Supplementary Figure S1B). The average tumor purity of PTs (46.6%) and CLMs (47.6%) was comparable (*P* = 0.94). Performing pairwise comparisons of sequencing reads obtained from a normal tissue, a PT, and an associated CLM tissue for each patient, we identified somatic mutations in the exomes and transcriptomes of PTs and CLMs (Supplementary Table S2, S3, and S4) and successfully confirmed a few mutations by Sanger sequencing (Supplementary Figure S2).

A recent report suggested that integrating exome-seq with RNA-seq improves the detection of cancer driver mutations [14]. Therefore, we examined whether variants excluded during the exome-seq mutation calling processes can be supported by RNA-seq. Among variants with ‘reject' calls by MuTect exome analysis, 137 mutations that had ‘Keep’ calls and more than 10 × coverage in MuTect RNA-seq analysis were revived (Supplementary Table S2). These included mutations in CRC-associated genes, such as *APC* and *PTEN*.

**Figure 1:**
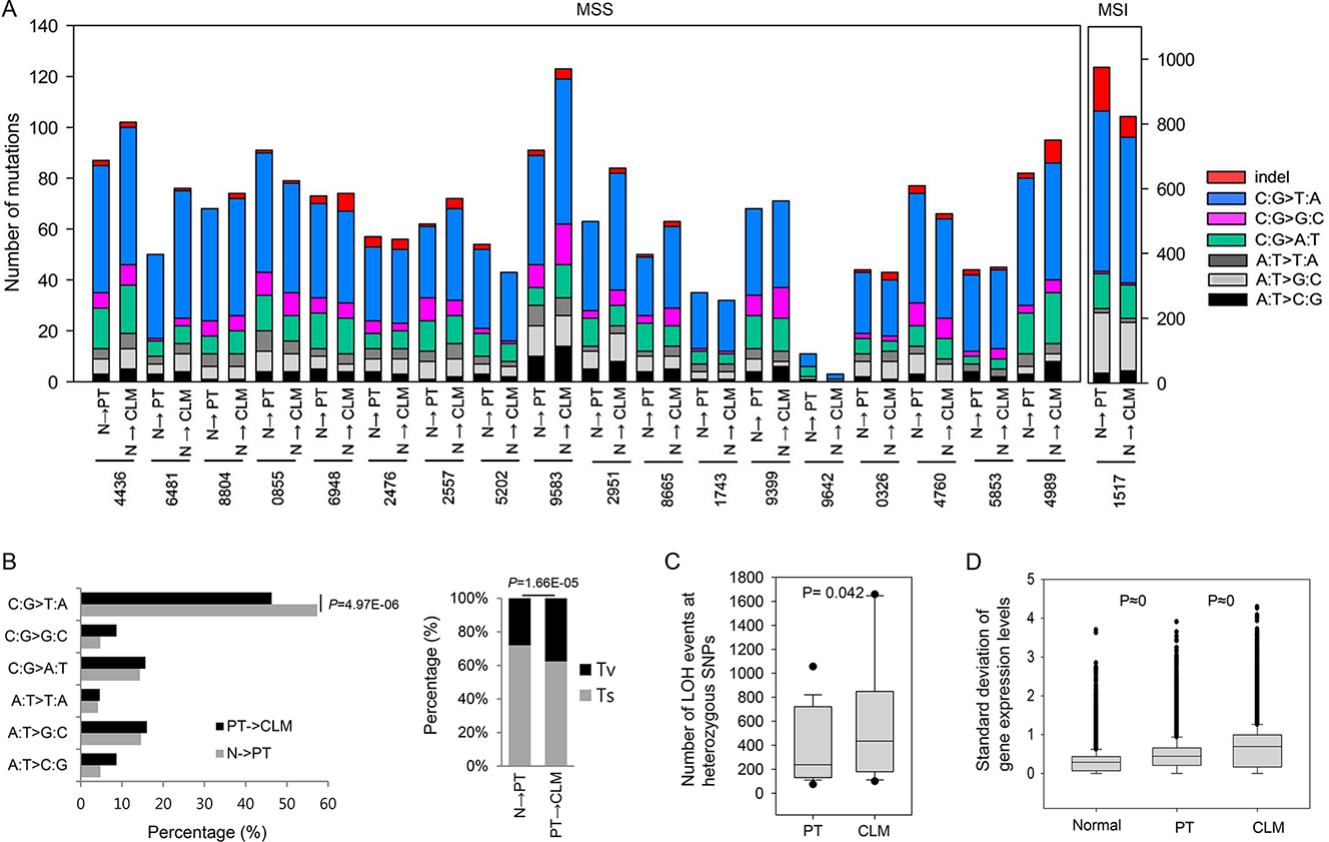
Molecular patterns of PTs and CLMs **A.** The number of mutations and base substitutions detected in 19 CRCs with CLMs. **B.** Percentage of base substitutions (left panel) and proportion of transversions (Tv) and transitions (Ts) (right panel) detected during the progression from normal tissues to PTs and ultimately to CLMs. **C.** Distribution of LOH counts occurring at heterozygote SNPs of PTs and CLMs. **D.** Distribution of the standard deviations of gene expression levels in normal tissues, PTs, and CLMs.

The average sequencing depth of exonic variants across all samples was ~101 ×, and the average nonsynonymous mutation rate was ~2.5 per megabase (Mb). Our cohort mostly consisted of microsatellite stable (MSS) CRC patients, with the exception of one MSI-high patient (Supplementary Table S1). The one MSI-high patient acquired an approximately 15-fold higher number of mutations than the rest (Figure 1A). The average number of mutations per MSS patient was 61.5 in the PTs and 66.7 in the matched CLMs (Figure 1A).

We observed high rates of C-to-T base substitutions in the PTs (57.4%), but the proportion significantly decreased during the progression from PTs to CLMs (46.3%) (Figure 1A and 1B). The decrease in C-to-T transitions occurred with a significant increase in transversions during the progression from PTs to CLMs (28.0% → 37.6%) (Figure 1B, right panel). Consistent with our data, a significant increase of transversions during the progression from PTs (33.5%) to CLMs (43.1%) was observed in an independent cohort of 15 CRCs (SRP034161, Supplementary Figure S3A). When 96 base substitutions were examined in a tri-nucleotide context, the proportion of mutations occurring in the context of G[C→A]C, T[C→A]C, T[C→G]T, A[A→C]G, G[C→T]C, A[C→T]C, and A[A→G]G was greater in CLMs than PTs (Supplementary Figure S3B), suggesting that the substitution pattern is slightly different between PTs and CLMs.

We assessed the genomic and transcriptomic variability during liver metastasis. When LOH occurring at heterozygote SNPs in tumors was examined as a sign of chromosomal aberrations, the number of LOH events was greater in CLMs than PTs (Figure 1C). In addition, we found that the gene expression deviations calculated based on RPKM gradually increased from normal tissues to PTs and, ultimately, to CLMs in a significant manner (Figure 1D).

### Classification of CRC mutations

**Figure 2:**
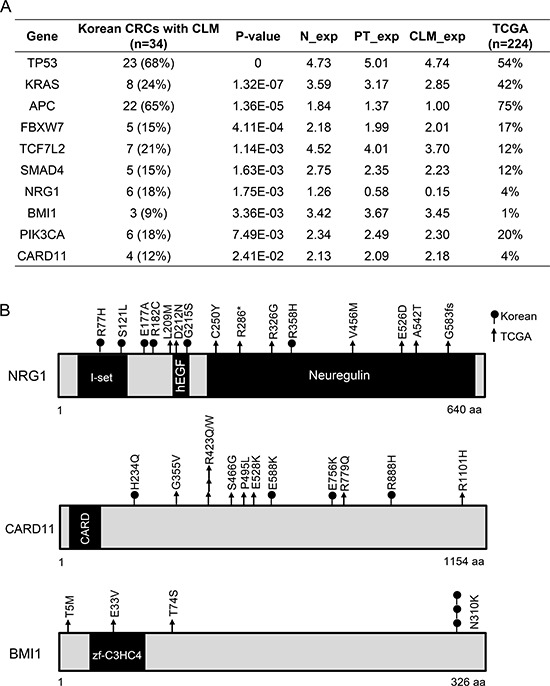
Significantly mutated genes in 34 Korean CRC patients with CLMs **A.** Significantly mutated genes analyzed by MutSig 1.4. **B.** *NRG1, CARD11*, and *BMI1* mutations found in Korean and TCGA CRC cohorts.

To identify significantly mutated genes in CRCs with CLMs, we analyzed the exomes of a total of 34 Korean CRCs with CLMs (See Method) using the following criteria: significantly mutated genes (*P* < 0.05) by MutSig analysis; genes mutated in at least three MSS patients; and genes expressed at more than 0.5 of the Log_2_ RPKM level in normal tissues, PTs, or CLMs. This analysis revealed 10 significantly mutated genes in CRCs with CLMs (Figure 2A). Consistent with previous genomic studies, CRCs with CLMs acquired frequent mutations in *APC, FBXW7, KRAS, PIK3CA, SMAD4, TCF7L2*, and *TP53* (Figure 2A). In addition, CRCs with CLMs significantly acquired mutations in *NRG1* (6 of 34 patients), *BMI1* (3 of 34 patients), and *CARD11* (4 of 34 patients) (Figure 2A and 2B). *NRG1* and *CARD11* were reported to be recurrently mutated in gastric cancer and B-cell lymphoma, respectively [15, 16]. In *BMI1*, an intestinal stem cell marker, we found a hotspot mutation (N310K) that is located at genomic position 22618420 on chromosome 10 (Figure 2B and Supplementary Figure S2). On the other hand, we could not identify significant CLM-specific mutations in 34 CLMs.

**Figure 3:**
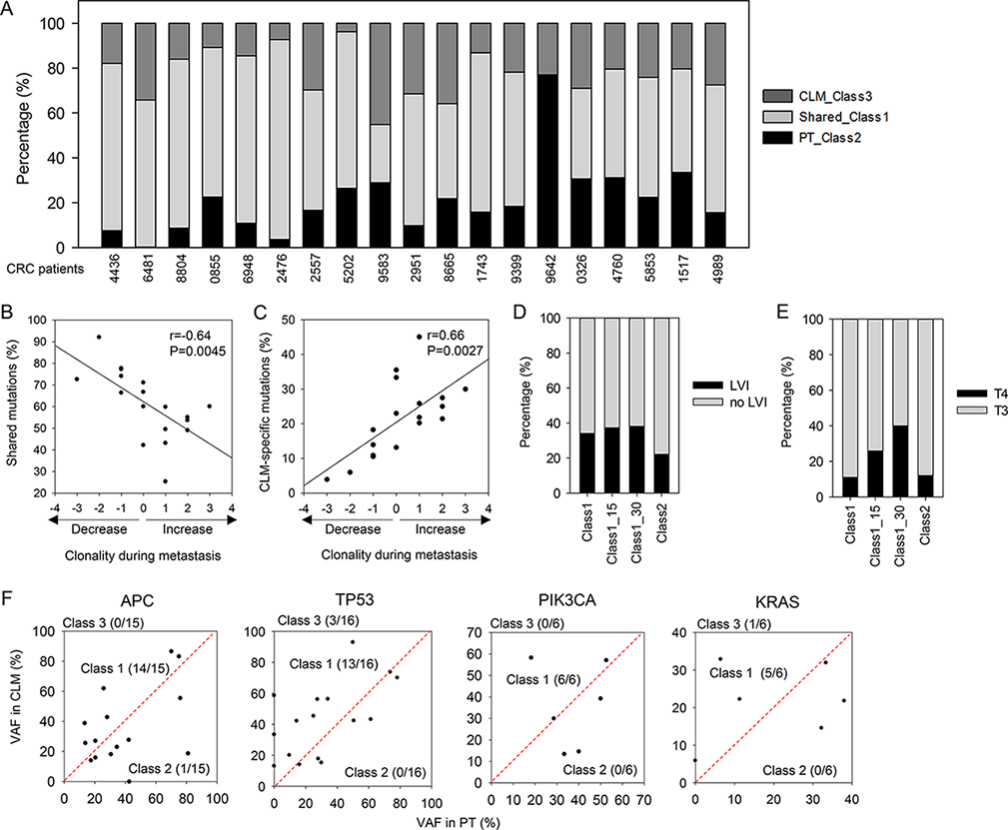
Mutational classes and their clinical associations **A.** Fraction of mutational classes in each patient. **B.** Correlation between clonality changes during metastasis and the proportion of shared mutations (Class 1). **C.** Correlation between clonality changes during metastasis and the proportion of CLM-specific mutations (Class 3). **D.** Percentage of Class 1 and 2 mutations detected in patients with or without LVI. Class 1_15 (30) indicates Class 1-H mutations that exhibit ≥ 15 (30)% higher VAFs in CLMs than PTs. **E.** Percentage of Class 1 and 2 mutations detected in patients in stage T3 or T4. **F.** Scatter plots for VAFs of CRC driver mutations presented as VAFs in PTs versus VAFs in CLMs.

Based on the absence or presence of mutations during liver metastasis, we classified mutations into three classes (Supplementary Table S5): (1) Class 1: mutations shared between PTs and CLMs, (2) Class 2: mutations detected in only PTs, and (3) Class 3: mutations detected in only CLMs. In our cohort, most of the mutations detected in PTs were concordantly detected in the matched CLMs, displaying the greatest proportion of Class 1 (~57.6%, Figure 3A). The proportion of Class 2 and Class 3 mutations was ~20.9% and ~21.5%, respectively (Figure 3A).

The proportion of Class 1 mutations was highly variable across patients (range from 25% to 92%, Figure 3A), indicating that clonal selection processes during metastasis across patients might be different. Therefore, we analyzed the clonality of the PTs and CLMs of each patient using SciClone analysis (Supplementary Figure S4). Notably, patients with decreased clonality during metastasis showed high mutational concordance between PTs and CLMs (Figure 3B). In contrast, increased clonality during metastasis was associated with low mutational concordance between PTs and CLMs and a high proportion of CLM-specific mutations (Figure 3B and 3C).

Then, we assessed whether Class 1 and Class 2 mutations exhibit distinct clinical association. The comparison of the two classes revealed that Class 1 mutations were more frequently detected in the patients with lymphovascular invasion (LVI), a phenomenon that is an indicator of poor prognosis as well as tumor cell intravasation into blood or lymphatic vessels [17]. The comparison revealed that 34.1% of Class 1 mutations were detected in patients with LVI, whereas 22.2% of Class 2 mutations were detected in the patients with LVI (Figure 3D, *P* = 1.98E-08). In particular, Class 1 mutations that exhibited higher VAFs in CLMs than PTs (hereafter referred to as Class 1-H) were detected in higher proportions in the patients with LVI (Figure 3D, 37.2% for 15% VAF difference, 38.2% for 30% VAF difference). Furthermore, Class 1-H mutations were more frequently detected in the patients with advanced tumor stages compared with Class 2 mutations (Figure 3E, *P* = 8.29E-07 for 15% VAF difference, *P* = 1.33E-08 for 30% VAF difference).

We also examined whether Class 1 and Class 2 mutations exhibited distinct biological association. Gene ontology analysis revealed that the gene set harboring Class 1 mutations significantly overrepresented the hallmarks of cancer [18], including cell proliferation, cell cycle, apoptosis, cell migration, tumor immunity, and epithelial-to-mesenchymal transition (Supplementary Table S6). Cancer-associated signaling pathways, including MAPK, WNT, and ERBB signaling, and well-known cancer genes involved in various cancer types were also overrepresented in the gene set harboring Class 1 mutations (Supplementary Table S6). Importantly, most mutations of the known CRC driver genes were shared between PTs and CLMs: 38 of 43 driver mutations from *APC, TP53, PIK3CA*, and *KRAS* were classified into Class 1 (Figure 3F). Only one *APC* mutation was PT-specific (Class 2), whereas one *KRAS* mutation and three *TP53* mutations were CLM-specific (Class 3) (Figure 3F).

### Co-occurrence of Class 1 mutations with chromosomal deletions in CLMs

**Figure 4:**
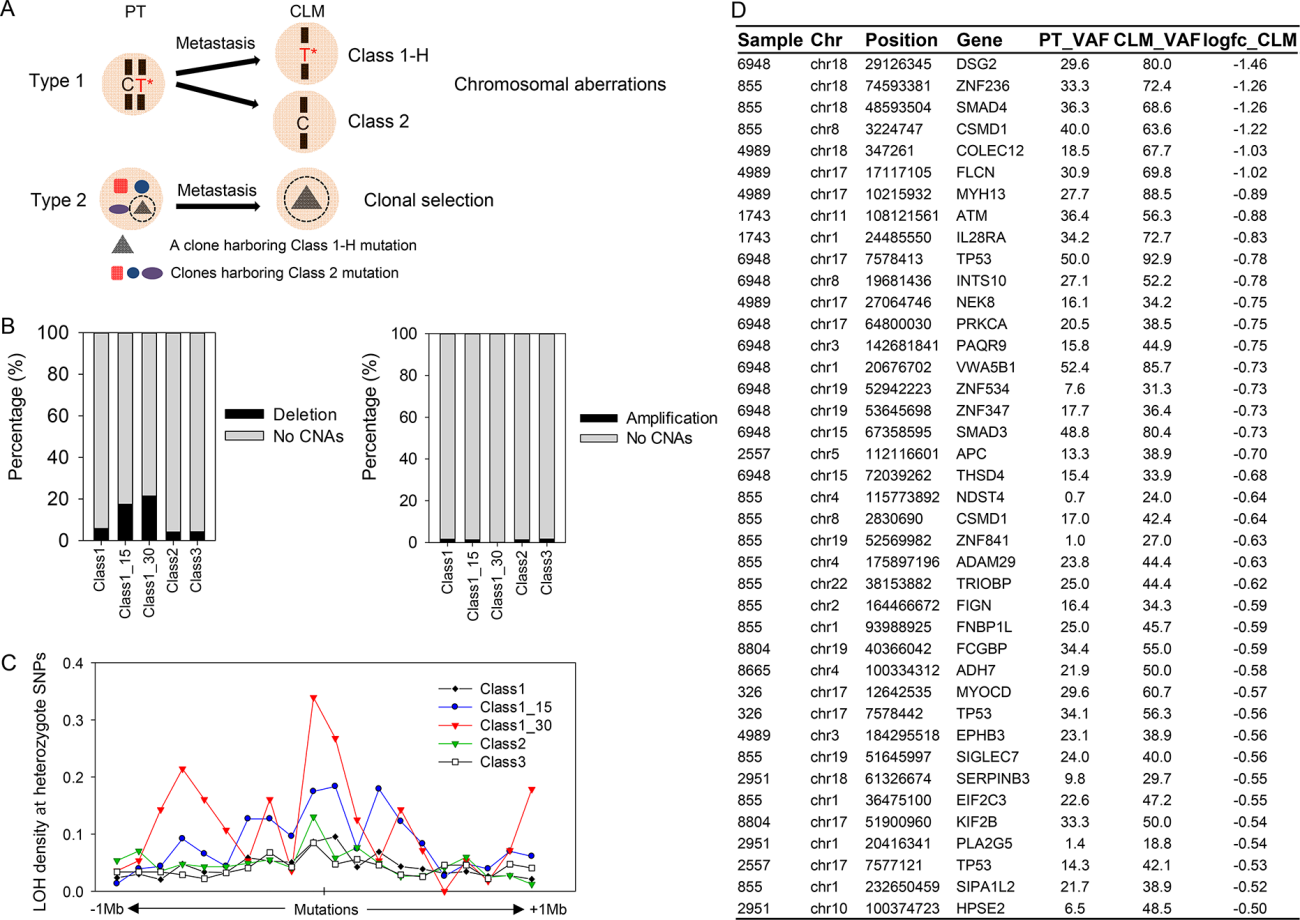
Co-occurrence of Class 1-H or Class 2 mutations with chromosomal aberrations **A.** Two types of molecular processes that potentially generate Class 1-H or Class 2 mutations. **B.** Frequency of the co-occurrence of chromosomal deletions (left) or amplification (right) with mutations. **C.** A LOH density plot presented as LOH counts at heterozygote SNPs per distance within ± 1-Mb regions relative to mutations. **D.** List of Class 1-H mutations superimposed with chromosomal deletions.

As presented in Figure 3D and 3E, Class 1-H mutations tended to be more significantly associated with the clinical characteristics of advanced cancer than were the Class 1 or Class 2 mutations. Based on this observation, we focused on the Class 1-H mutations that exhibit ≥ 15% or ≥ 30% higher VAFs in CLMs than in PTs. Class 1-H and Class 2 mutations may occur through various molecular processes. Assuming that a mutation occurs at a given genomic position in a PT, a subsequent deletion occurring at the locus during metastasis would generate Class 1-H or Class 2 mutations (Figure 4A, Type 1). Alternatively, clonal selection during metastasis would also generate Class 1-H or Class 2 mutations (Figure 4A, Type 2).

To assess the association of Class 1-H and Class 2 mutations with chromosomal aberrations, we examined the co-occurrence of Class 1-H or Class 2 mutations with copy number alterations (CNAs). Here, the copy number of the chromosomal regions was estimated from the exome-seq data using VarScan 2, and the chromosomal regions that deviated by more than *a* ± 0.5-fold-change (log_2_ ratio) from the normal chromosomal counts were selected as tumor CNAs. Examination of the co-occurrence of mutations with CNA regions revealed that Class 1 mutations occurred more frequently with chromosomal deletions (5.80%) than Class 2 (4.13%) or Class 3 mutations (4.23%) (Figure 4B, left panel). Remarkably, Class 1-H mutations were more frequently superimposed with chromosomal deletions than with the other mutation classes (Figure 4B, left panel, 17.47% for 15% VAF difference, 21.43% for 30% VAF difference). However, this phenomenon was not observed in the chromosomal amplification regions (Figure 4B, right panel).

We also analyzed the LOH density at nearby heterozygote SNPs around mutations as an indicator of chromosomal aberrations. The examination of ± 1-Mb regions relative to the mutations revealed a higher LOH density in regions encompassing Class 1-H mutations than in the other classes (Figure 4C). Importantly, many Class 1-H mutations superimposed with chromosomal deletions were found in the known tumor suppressors, including *APC, TP53, SMAD4*, and *ATM*, and the CRC-associated genes, including *CSMD1* [19], *FLCN* [20], and *DSG2* [21] (Figure 4D). Among them, multiple mutations in *CSMD1* and *TP53* co-occurred with chromosomal deletions (Figure 4D), suggesting that both mutations and chromosomal deletions may occasionally inactivate both alleles of these genes, as proposed by Knudson's two-hit hypothesis [22]. Supporting the importance of *CSMD1* mutations, TCGA CRC patients harboring *CSMD1* mutations had poorer survival rates than patients without *CSMD1* mutations (Supplementary Figure S5). Among the candidate genes (Figure 4D), *FLCN* was recently reported to be a recurrently mutated gene in an African American CRC cohort [20].

### Selective expression of mutant alleles in the transcriptomes of CLMs

**Figure 5:**
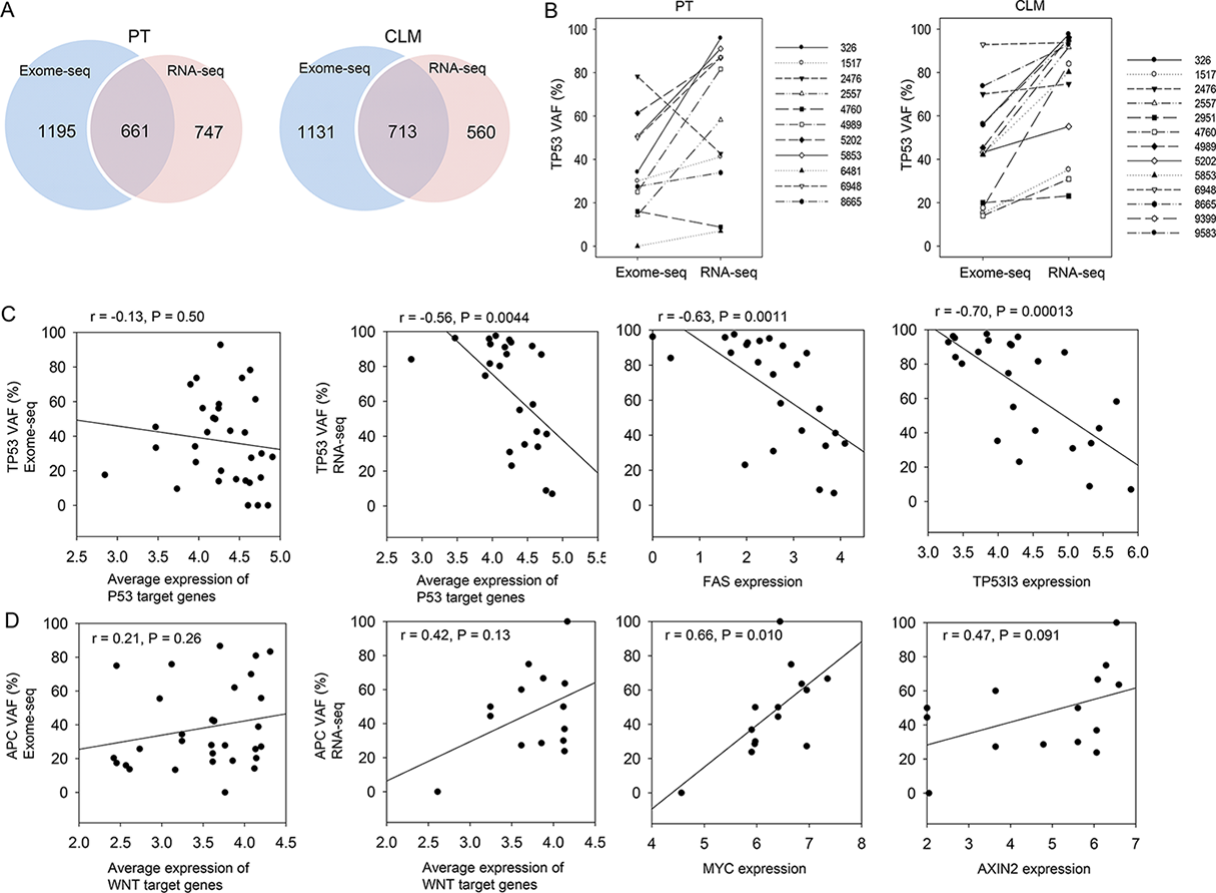
Integration of exome-seq with RNA-seq and functional implication of selective expression of mutant alleles **A.** Intersection of exome-seq and RNA-seq mutations. **B.** VAFs of *TP53* mutations in the exomes and transcriptomes of PTs and CLMs. **C.** Correlation between *TP53* VAFs and the expression level of p53-target genes (*BBC3, BAX, FAS, APAF1, CCNG1, CDKN1A, GADD45A, PTEN, SFN, TSC2*, and *TP53i3*). **D.** Correlation between *APC* VAFs and the expression level of WNT-target genes (*MYC, CCND1, HNF1A, LEF1, PPARD, JUN, FOSL1, MMP7, AXIN2, NRCAM, TCF4, CLDN1, VEGFA, FGF18, MYCBP, ID2, TERT, LGR5*, and *FZD7*).

We compared mutation profiles in the exomes and transcriptomes of PTs and CLMs. The intersection of exome-seq and RNA-seq revealed that 661 mutations in PTs (36% of exome-seq mutations, 47% of RNA-seq mutations) and 713 mutations in CLMs (39% of exome-seq mutations, 56% of RNA-seq mutations) were common between the two data sets (Figure 5A). The comparison also presented the discordance between exomes and transcriptomes. According to our analysis, exome-seq specific mutations were mainly generated due to the low RNA expression of mutated genes: ~65% of genes harboring exome-seq-specific mutations were estimated as below 1 (log_2_ RPKM) in normal samples, PTs, and CLMs (Supplementary Figure S6A and S6B). On the other hand, RNA-seq-specific mutations might be derived from the low coverage of the mutations in exome-seq (Supplementary Figure S6C, ~18% in our study) or RNA editing.

Recently, the integration of exome-seq with RNA-seq revealed that the VAFs of oncogenic driver mutations were higher in transcriptomes than in exomes [14]. To extend this observation, we compared the mutational VAFs detected from the exomes and transcriptomes. This analysis identified 89 mutations that exhibit ≥ 15% higher VAFs in transcriptomes than in exomes in both PTs and CLMs (Supplementary Table S7). The list contained several genes responsible for CRC development or metastasis, including *BMI1* [23], *HOXB9* [24], *PLXNB1* [25], *POLD1* [26], *TGIF1* [27], *SOX9* [28], as well as *TP53* (Supplementary Table S7). Remarkably, the VAFs of the *TP53* mutations were higher in the transcriptomes of PTs and CLMs than in their exomes, except for two mutations in PTs (Figure 5B). Notably, *TP53* VAFs from six CRC patients (326, 2557, 9399, 5853, 4989, and 8665) were ~50% in the exomes of CLMs, whereas the VAFs were up to ~100% in the transcriptomes of CLMs (Supplementary Figure S7), suggesting the selective expression of *TP53* mutant alleles in CLMs.

Next, we assessed whether the selective expression of mutant alleles has functional association. Given that *TP53* mutations can lead to the decreased expression of p53-target genes, we expected a negative correlation between *TP53* VAFs and p53-target gene expression levels. As expected, the correlation analysis revealed that the average expression level of 11 p53-target genes obtained from the KEGG pathway displayed a significant negative correlation with *TP53* VAFs from RNA-seq (Figure 5C). However, this phenomenon was not observed with *TP53* VAFs from exome-seq (Figure 5C). For the *APC* mutations, we expected a positive correlation between the *APC* VAFs and WNT-target gene expression levels because *APC* is a negative regulator of the WNT signaling pathway. Indeed, the *APC* VAFs from RNA-seq exhibited a stronger positive correlation with the average expression level of 19 WNT-target genes than those from exome-seq (Figure 5D). The expression level of *MYC*, a well-characterized WNT-target, showed a significant positive correlation with the *APC* VAFs from RNA-seq (Figure 5D). These results might suggest the functional implication for the selective expression of mutant alleles at the transcriptome level.

### Candidate CLM mutations enriched in the transcriptome of CLMs

**Figure 6:**
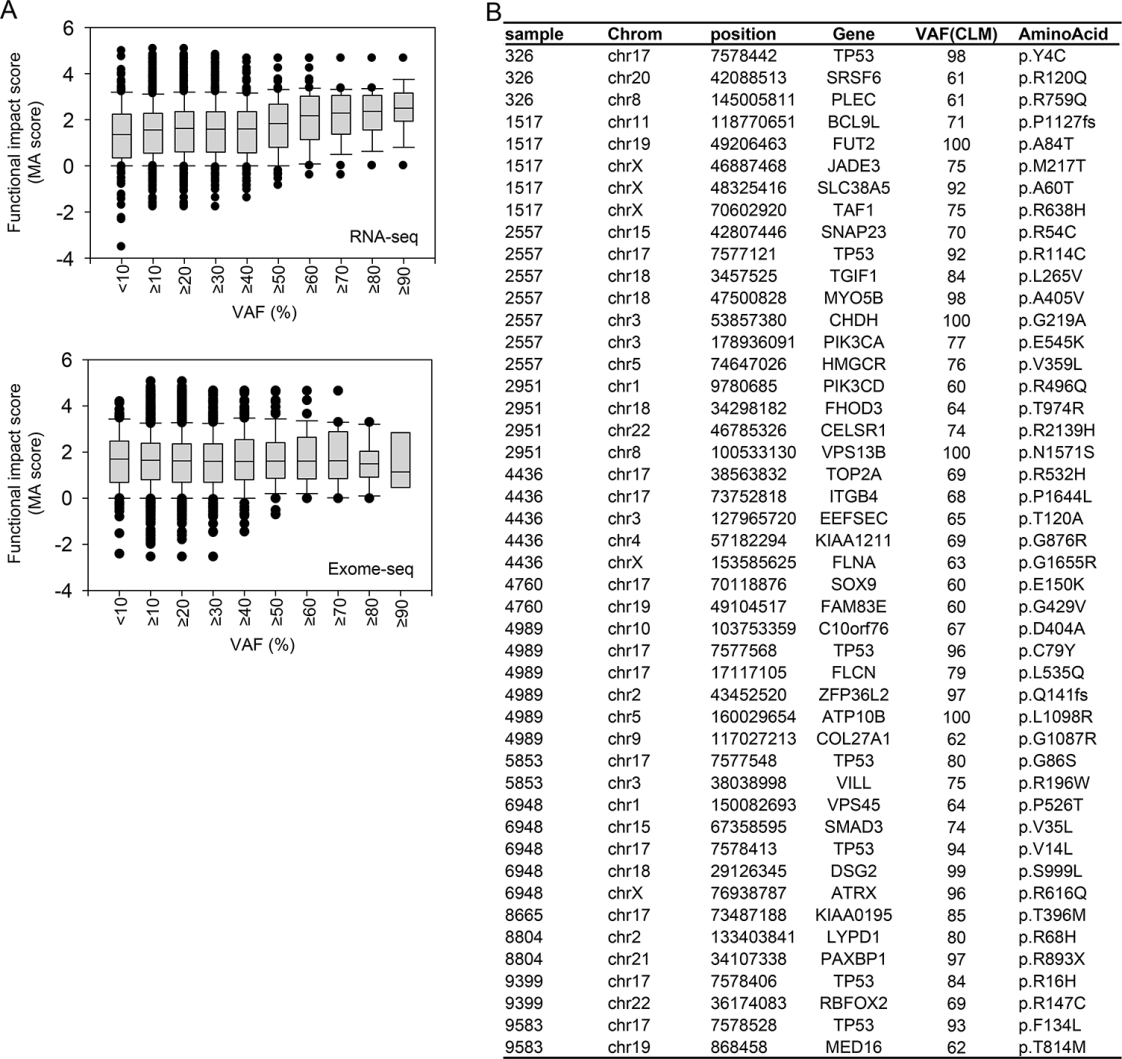
Enrichment of mutations exhibiting high functional impact in the transcriptomes of CLMs **A.** Distribution of mutational functional impact scores according to increasing VAFs in RNA-seq (upper) and exome-seq (bottom) analysis of CLMs. **B.** Candidate CLM mutations enriched in the transcriptomes of CLMs.

In general, mutations exhibiting strong functional effects may have a high chance of being negatively or positively selected during tumor progression. Therefore, we explored whether mutations having a high functional impact are enriched in CLMs during metastasis as a consequence of positive selection. Scoring the functional impact of the mutations by Mutation Assessor (MA) [29], we found that mutations exhibiting higher MA scores tended to be detected with higher VAFs in the transcriptome of CLMs (Figure 6A, upper panel). However, the phenomenon was not observed in the exomes of CLMs (Figure 6A, bottom panel).

Figure 6A (upper panel) demonstrates that mutations exhibiting ≥ 60% VAFs in CLMs displayed a distinctly high average MA score (greater than 2), which typically indicates ‘functional mutations’ [29]. Based on this observation, we selected candidate CLM mutations that satisfy the following conditions: mutations that exhibit ≥ 60% VAFs in CLMs and ≥ 15% higher VAFs in the transcriptomes than in the exomes of PTs or CLMs. The list contained up to seven *TP53* mutations (Figure 6B), indicating the functional relevance of *TP53* mutations in CLMs. The list also included a substantial number of mutations located in well-established cancer- or metastasis-associated genes (Figure 6B): *BCL9L* [30], *DSG2, FLCN, FLNA* [31], *ITGB4* [32], *PIK3CA, PIK3CD* [33], *PLEC* [34], *SMAD3, SOX9, SRSF6* [35], *TGIF1*, and *TOP2A* [36].

### Splicing events occurring in CRCs

To further assess the functional mutations by integrating the exomes and transcriptomes, we analyzed mutation-dependent splicing events in CRCs. Forty-two mutations were annotated as 5′ or 3′ splice-site mutations from the exome-seq data (Supplementary Table S8), and exon–exon junction reads, which may indicate branches between exons by splicing, were selected and counted from the RNA-seq data (data not shown). The integration of both data sets revealed that 11 of the 42 splice-site mutations might change the splicing patterns in PTs and CLMs (indicated in red in Supplementary Table S8).

**Figure 7:**
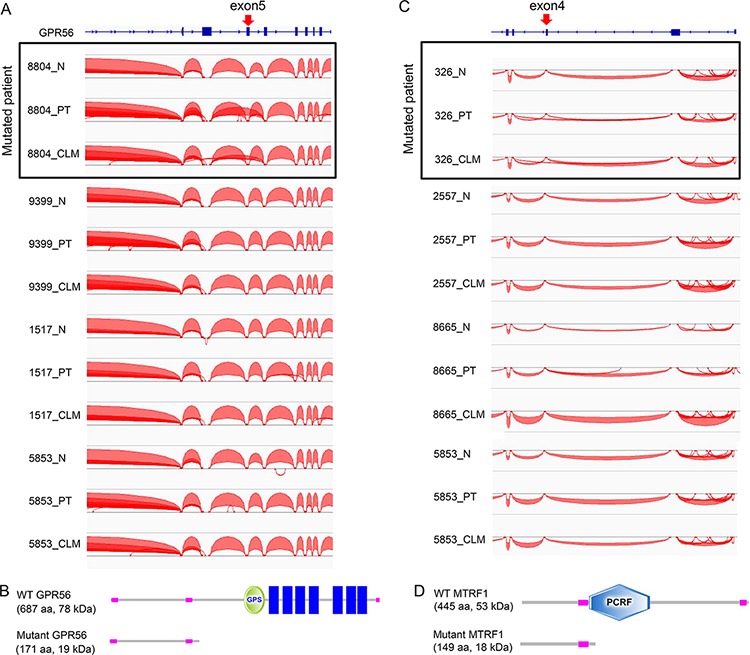
Mutation-dependent splicing events occurring in CRCs **A.** Exon 5 skipping associated with a *GPR56* splice site mutation in patient 8804. Curved lines between exons indicate exon-exon junction reads. An arrow denotes the position of a splice-site mutation. **B.** A predicted GPR56 protein generated by a *GPR56* splice-site mutation. **C.** Exon 4 skipping associated with a *MTRF1* splice site mutation in patient 326. **D.** A predicted MTRF1 protein generated by a *MTRF1* splice site mutation.

For instance, a *GPR56* mutation at a 5′ splice-site adjacent to exon 5 (exon 5:c.620 + 1G > A in NM_005682) correlated with exon 5 skipping in patient 8804, who harbored the mutation, but not in other patients lacking the mutation (Figure 7A). Because exon 5 skipping by the mutation may give rise to a premature stop codon that abrogates all functional domains of GPR56 (Figure 7B), which has been known to play a tumor-suppressive role [37], the *GPR56* splice-site mutation is likely involved in CRC development. We also found a *MTRF1* splice-site mutation (exon 4:c.416–2A > T in NM_004294) in patient 326, which correlated with exon 4 skipping and may introduce a premature stop codon (Figure 7C and 7D). Splice-site mutations in the cancer-associated genes *RNF31* [38] and *ATM* [39] also correlated with the skipping of exons harboring these mutations and may lead to a partial deletion of these proteins (Supplementary Figure S8A and S8B).

## DISCUSSION

By combining two methods (exome-seq and RNA-seq) with two different types of tumor samples (PTs and CLMs), this study demonstrated the mutation profiles of PTs and CLMs at the exome and transcriptome levels. In this study, we used VAF as a parameter that depends on mutation prevalence, genetic heterogeneity, and copy-number alterations. Because VAF also highly depends on normal cell contamination, we used high-purity tumors to minimize the interference from normal cell contamination.

PTs and CLMs displayed different global molecular patterns, including base substitutions, LOHs at heterozygote SNPs, and gene expression variability. Nonetheless, they shared the majority of mutations, indicating the common clonal origin of PTs and CLMs. This phenomenon was apparent in driver mutations in *APC, KRAS, PIK3CA*, and *TP53*. This is because the driver mutations that occur in the early stages of CRC development may expand through sustained tumor growth [9]. Notably, the proportion of shared mutations between PTs and CLMs was highly correlated with clonality change during metastasis. Increased clonality during metastasis, which may indicate clonal diversification, was associated with a low mutational concordance between PTs and CLMs, whereas decreased clonality during metastasis, which may indicate convergent clonal transmission, was associated with high mutational concordance between PTs and CLMs.

Tumor clones compete with each other within heterogeneous tumor populations, thus resulting in the positive selection of fitter clones harboring advantageous mutations during metastasis [40]. Therefore, ubiquitous detection of Class 1 mutations, even after metastases, might indicate increased fitness for liver metastasis compared with Class 2 mutations, which are absent after metastases. Supporting this hypothesis, Class 1 contained a larger proportion of mutations associated with the traits of advanced cancer, including advanced tumor stages or LVI, compared with Class 2 mutations. Moreover, genes harboring Class 1 mutations were significantly associated with the hallmarks of cancer and the known CRC drivers were classified into Class 1.

Consistent with a previous report [7], we could not identify significant CLM-specific mutations. Instead, we found various CRC-associated genes harboring PT-derived mutations by multiple analyses: significantly mutated genes (*TP53, KRAS, APC, FBXW7, TCF7L2, SMAD4, PIK3CA, NRG1, BMI1*, and *CARD11*), mutations superimposed with chromosomal deletions (*APC, ATM, CSMD1, DSG2, FLCN, SMAD4*, and *TP53*), mutations exhibiting greater VAFs in transcriptomes than in exomes (*BMI1, HOXB9, PLXNB1, POLD1, TGIF1, SOX9*, and *TP53*), and mutations enriched in the transcriptomes of CLMs (*BCL9L, DSG2, FLCN, FLNA, ITGB4, PIK3CA, PIK3CD, PLEC, SMAD3, SOX9, SRSF6, TGIF1, TOP2A*, and *TP53*). In particular, *BMI1, DSG2, FLCN, SOX9*, and *TGIF1* were discovered to be candidate CRC genes in multiple analyses described above. *BMI1* maintains self-renewal and the tumorigenic potential of CRC stem cells [41]. *DSG2* is involved in CRC tumorigenesis by activating EGFR signaling [21]. *FLCN* has been shown to be a causal gene for Birt-Hogg-Dubé syndrome, which is associated with an increased risk for renal or colorectal cancers [42]. Importantly, *FLCN* was recently reported to be a novel recurrently mutated gene in an African American CRC cohort [20]. *SOX9* was identified to be a frequently mutated gene in a TCGA CRC cohort [3]. TGIF1 was degraded by the CRC tumor suppressor FBXW7 and enhanced TGFβ-dependent cell growth and migration [27]. Therefore, further studies are required to demonstrate the metastatic roles of these mutated genes.

In this study, there are several limitations, including small sample sizes and the lack of functional validation. Nonetheless, our results suggest that the mutation profiles of PTs and CLMs at the exome and transcriptome levels may be valuable for understanding the underlying molecular alterations in CRCs. Further studies employing large cohorts followed by functional assessment will enable the discovery of useful therapeutic targets against CLMs.

## MATERIALS AND METHODS

### Patients

Nineteen CRC patients with CLMs were recruited from Asan Medical Center (Korea) with informed consent. A total of 57 tissues were freshly resected from normal colorectal tissues, PTs, and the associated CLMs of the patients. Normal tissues were collected at areas > 5 cm from the tumor margin by sub-epithelial dissection (at least 95% epithelial cells on histologic examination), and PTs and CLMs were synchronously collected. To improve the efficiency of mutation detection, we used PTs and CLMs containing approximately > 90% tumor cells under triplicate histological reviews (Supplementary Figure S1A). Our 19 CRC patients were supplemented with 15 CRC patients (SRP034161) [13], constituting a total of 34 Korean CRC patients with CLMs. This study was approved by the Institutional Review Board (No.2014–0150).

### Whole-exome sequencing and data analysis

Genomic DNA was extracted using the Puregene^TM^ DNA purification kit (Qiagen, Venlo, Netherlands). Libraries were constructed using the Illumina TruSeq DNA Sample Prep Kit (San Diego, CA, USA), and exome enrichment was performed using the SeqCap EZ Human Exome Library v2.0 kit (Roche NimbleGen, Madison, WI, USA). After the quantity of the libraries was assessed by the CFX96 real-time system (Bio-Rad, Hercules, CA), paired-end sequencing was performed using the Illumina HiSeq 2000 sequencing system. The resulting FASTQ sequencing read files were aligned on the reference human genome 19 (hg19) using the Burrows-Wheeler Aligner [43]. Using Picard (Broad Institute), the SAM files were sorted and converted into BAM files, and duplicate reads were removed. Then, the remaining reads were processed using the Genome Analysis Toolkit (GATK) to generate realigned BAM files [44]. MuTect was used for the highly sensitive detection of somatic single-nucleotide variants (sSNVs) [45], and Strelka was used to detect insertion/deletion variants (indels) [46]. VarScan 2 was used to identify CNAs and to determine loss of heterozygosity (LOH) at heterozygote dbSNPs [47]. MutSig 1.4 (Broad Institute) was used to identify the significantly mutated genes, and SciClone was applied to infer tumor clonality [48]. ASCAT v2.1 analysis was performed to estimate tumor purity [49]. All programs were run under the default parameter settings. Coding variants were selected by annotation using dbNSFP [50] and ANNOVAR [51], and non-pathogenic dbSNPs (dbSNP version 132) were further filtered from the list.

### RNA sequencing and data analysis

After isolating the total RNA using the RNeasy^®^ Mini kit (Qiagen), libraries were constructed using the Illumina TruSeq RNA Sample Prep Kit v2 and sequenced using the Illumina HiSeq 2000. The reads were mapped on hg19 using TopHat v2.0.6 [52]. For detection of mutations, after removing duplicates using Picard, the resulting BAM files were subjected to MuTect and Strelka analyses. For estimation of the transcript expression levels, we calculated the reads per kilobase per million mapped reads (RPKM) using custom Python scripts. For detection of splicing events, the exon–exon junction reads were detected and counted using custom JAVA scripts.

### Sanger sequencing

To validate mutations, genomic regions containing mutations were amplified with primers (Supplementary Table S9) using the GeneAmp PCR System 9700 (Thermo Fisher Scientific, Waltham, MA). The PCR conditions were as follows: 95°C for 3 min, 35 cycles of 95°C for 20 s, 60°C for 20 s, and 72°C for 1 min. Sanger sequencing was performed by GENOTECH (Deajeon, Korea).

### Statistical analysis

Student's *t*-test, the chi-square test, or linear regression was applied to test significant differences or correlations between two groups using SPSS v11.5 (SPSS Inc., IL, USA). *P* < 0.05 was considered statistically significant.

### Data access

Sequencing data are available from the NCBI (http://www.ncbi.nlm.nih.gov/) via the accession numbers SRP041725 (exome-seq) and GSE50760 (RNA-seq).

## SUPPLEMENTARY FIGURES AND TABLES





## Abbreviations

CLM: colorectal cancer liver metastasis
CNA: copy-number alteration
CRC: colorectal cancer
Exome-seq: exome sequencing
LOH: loss of heterozygosity
LVI: lymphovascular invasion
MA: mutation assessor
MSI: microsatellite instability
MSS: microsatellite stable
PT: primary tumor
RNA-seq: RNA sequencing
RPKM: reads per kilobase per million mapped reads
SNP: single-nucleotide polymorphism
sSNV: somatic single-nucleotide variant
TCGA: the Cancer Genome Atlas
VAF: variant allele frequency
