# Supplementary material for: Genome-wide mutation profiles of colorectal tumors and associated liver metastases at the exome and transcriptome levels
Source: Oncotarget. 2015 Jun 1;6(26):22179–90. doi: 10.18632/oncotarget.4246 (PMC4673155; doi:10.18632/oncotarget.4246)
Supplement: Supplementary file 1 [file oncotarget-06-22179-s001.pdf]

SUPPLEMENTARY FIGURES AND TABLES

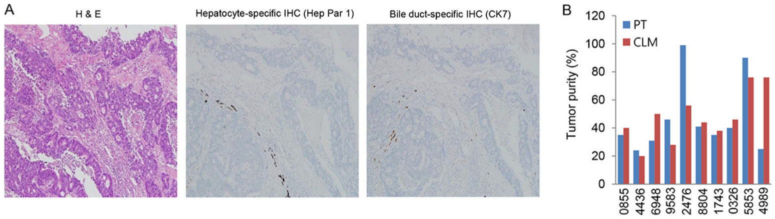

Supplementary Figure S1: A. Representative images for histological examination of tumor cell contents in a CLM tissue (×100 magnified images). Immunohistochemistry revealed the low amounts of normal hepatocytes and bile duct cells, indicating the high tumor purity. Left, Hematoxylin and eosin stain; Middle, hepatocyte-specific immunohistochemistry (staining by Hep Par 1 antibody), Right, bile duct-specific immunohistochemistry (staining by CK7 antibody). **B.** Tumor purity estimated mathematically using ASCAT v2.1. Tumor purity of several samples, which was unable to be analyzed using ASCAT v2.1, was excluded.

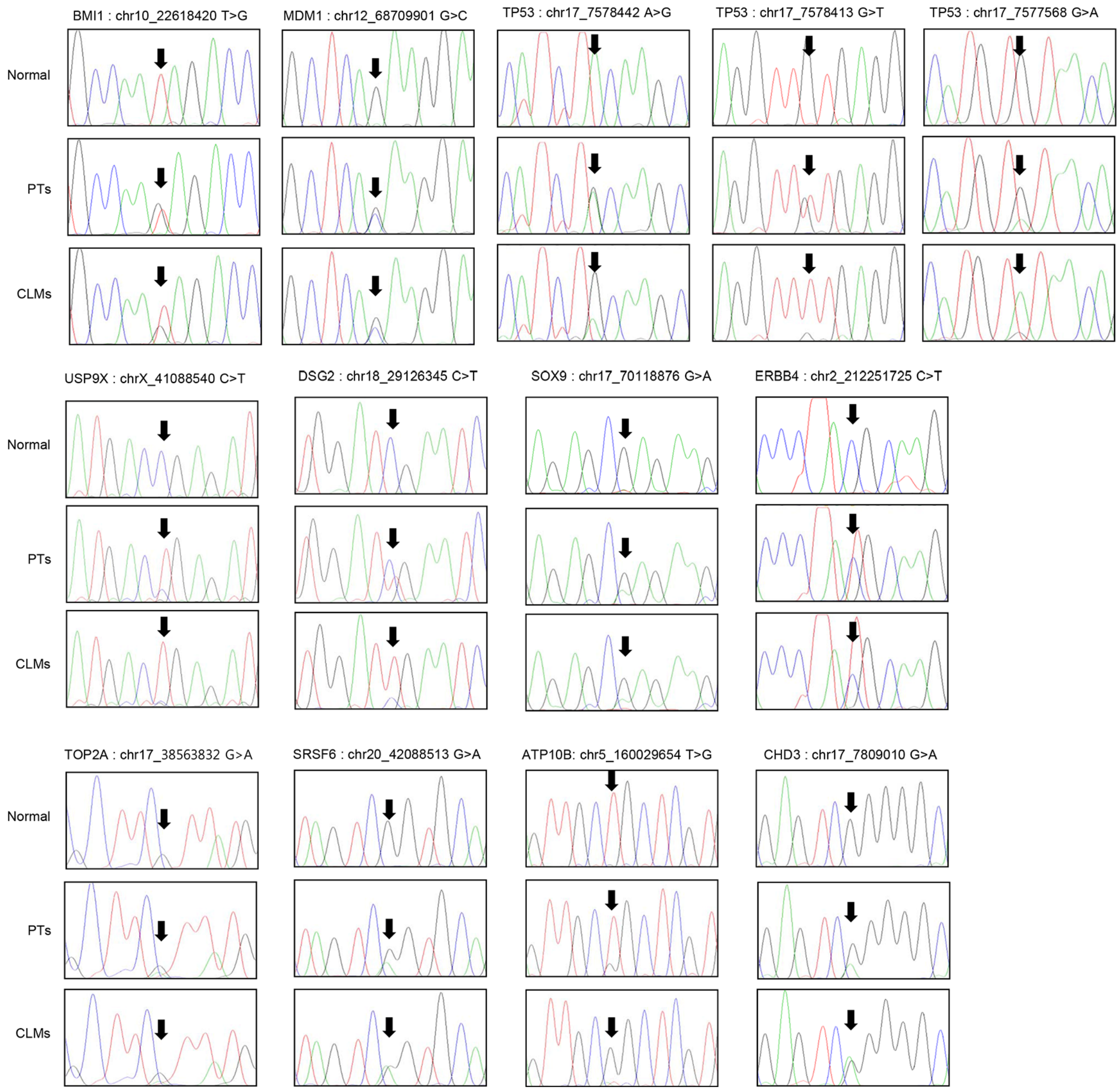

Supplementary Figure S2: Validation of somatic mutations by Sanger sequencing.

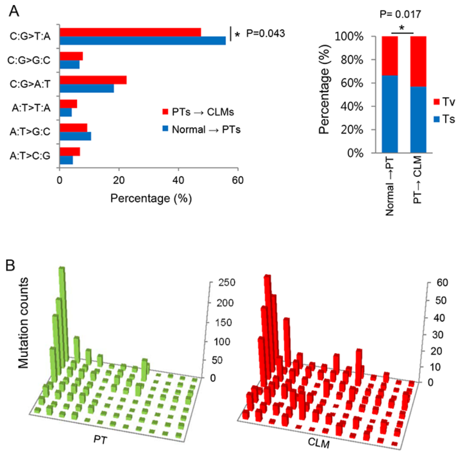

Base substitutions at tri-nucleotide context

G[C->T]G	G[C->T]C	G[C->T]A	G[C->T]T	G[C->A]G	G[C->A]C	G[C->A]A	G[C->A]T	G[C->G]G	G[C->G]C	G[C->G]A	G[C->G]T
C[C->T]G	C[C->T]C	C[C->T]A	C[C->T]T	C[C->A]G	C[C->A]C	C[C->A]A	C[C->A]T	C[C->G]G	C[C->G]C	C[C->G]A	C[C->G]T
A[C->T]G	A[C->T]C	A[C->T]A	A[C->T]T	A[C->A]G	A[C->A]C	A[C->A]A	A[C->A]T	A[C->G]G	A[C->G]C	A[C->G]A	A[C->G]T
T[C->T]G	T[C->T]C	T[C->T]A	T[C->T]T	T[C->A]G	T[C->A]C	T[C->A]A	T[C->A]T	T[C->G]G	T[C->G]C	T[C->G]A	T[C->G]T
G[A->G]G	G[A->G]C	G[A->G]A	G[A->G]T	G[A->C]G	G[A->C]C	G[A->C]A	G[A->C]T	G[A->T]G	G[A->T]C	G[A->T]A	G[A->T]T
C[A->G]G	C[A->G]C	C[A->G]A	C[A->G]T	C[A->C]G	C[A->C]C	C[A->C]A	C[A->C]T	C[A->T]G	C[A->T]C	C[A->T]A	C[A->T]T
A[A->G]G	A[A->G]C	A[A->G]A	A[A->G]T	A[A->C]G	A[A->C]C	A[A->C]A	A[A->C]T	A[A->T]G	A[A->T]C	A[A->T]A	A[A->T]T
T[A->G]G	T[A->G]C	T[A->G]A	T[A->G]T	T[A->C]G	T[A->C]C	T[A->C]A	T[A->C]T	T[A->T]G	T[A->T]C	T[A->T]A	T[A->T]T

Supplementary Figure S3: Molecular patterns identified in CRC patients with CLMs. A. Percentage of base substitutions (left), and proportion of transversion (Tv) and transition (Ts) mutations (right) analyzed using exome-seq data from publicly available independent CRC patients. **B.** 96 base substitutions identified in 19 CRC patients with CLM.

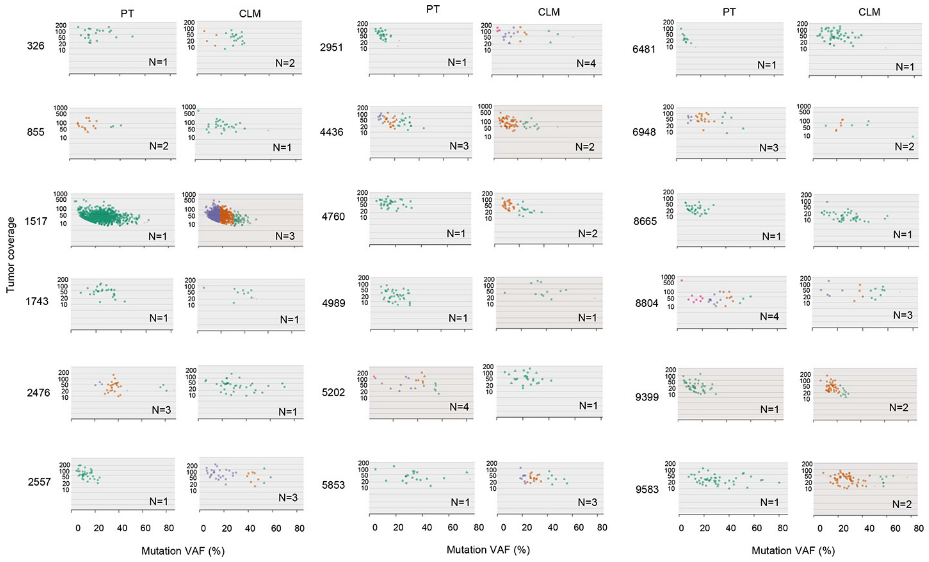

Supplementary Figure S4: The Clonality of PTs and CLMs analyzed by SciClone. Mutations that are located within genomic loci with copy number 2 were used to analyze clonality. Number N indicates the analyzed clonal count of each sample. Different colors indicate clusters of clones.

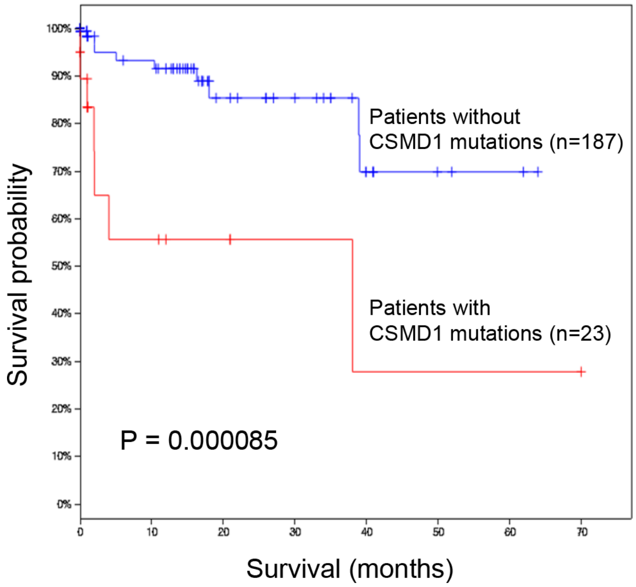

Supplementary Figure S5: A Kaplan-Meier survival curve analyzed according to the absence or presence of mutations in *CSMD1* using the TCGA CRC dataset. Log rank *P*-value is shown.

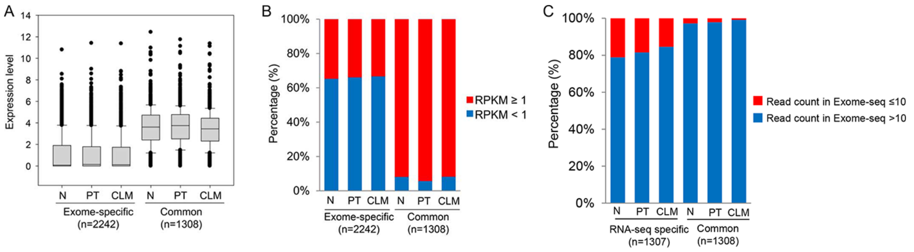

Supplementary Figure S6: A, B. The expression level of genes harboring exome-seq specific or common mutations (mutations overlapped in exome-seq and RNA-seq data). C. Read depth of RNA-seq specific or common mutations in exome-seq.

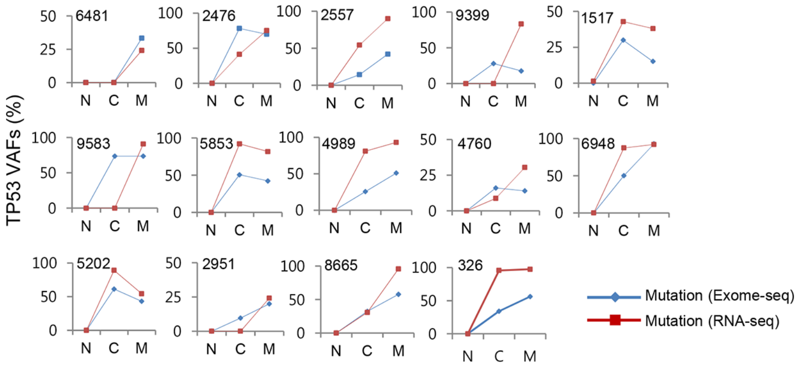

Supplementary Figure S7: The VAFs of *TP53* mutations in exomes and transcvriptomes of normal (N), PTs (C), and CLMs (M).

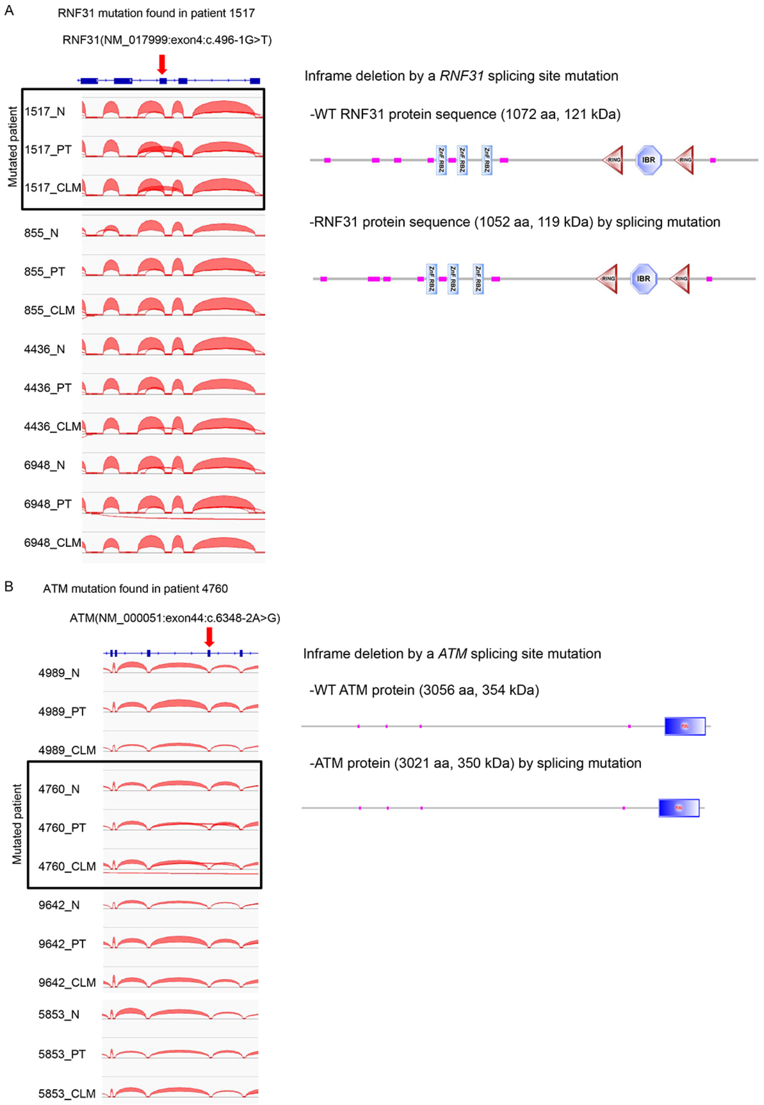

Supplementary Figure S8: Mutation-dependent splicing events in CRCs. A. Exon 4 skipping by a *RNF31* splice site mutation in patient 1517. **B.** Exon 44 skipping by an *ATM* splice site mutation in patient 4760.

Supplementary Table S1: Clinical information of 19 CRC patients with CLMs.

Supplementary Table S2: sSNVs identified from 19 CRC patients with CLMs.

Supplementary Table S3: Insertion-deletion mutations identified from 19 CRC patients with CLMs.

Supplementary Table S4: Somatic mutations identified from RNA-seq of 19 CRC patients with CLMs.

Supplementary Table S5: Three classification mutations identified in 19 CRC patients with CLMs.

Supplementary Table S6: Biological processes overrepresented in three classification mutations.

Supplementary Table S7: Somatic mutations exhibiting differential VAFs between exome and transcriptome.

Supplementary Table S8: Splice site mutations found in 19 CRC patients with CLMs.

Supplementary Table S9: Primers for Sanger sequencing.
